# Complete resection of an intrapericardial bronchogenic cyst with median sternotomy showing high CA19-9 level fluid sampling by EBUS-TBNA: a case report

**DOI:** 10.1186/s44215-022-00021-2

**Published:** 2023-02-14

**Authors:** Hirotoshi Suzuki, Itaru Ishida, Satoshi Kawatsu, Yuyo Suzuki, Katsuhiko Oda, Hiroyuki Oura

**Affiliations:** 1 Department of Thoracic Surgery, Iwate Prefectural Central Hospital, 1-4-1 Ueda, Morioka, Iwate, 020-0066 Japan; 2 Department of Cardiovascular Surgery, Iwate Prefectural Central Hospital, 1-4-1 Ueda, Morioka, Iwate, 020-0066 Japan

**Keywords:** Intrapericardial bronchogenic cyst, Cardiopulmonary bypass, Endobronchial ultrasonography transbronchial needle aspiration

## Abstract

**Background:**

An intrapericardial bronchogenic cyst (IBC) is rare and compresses the surrounding organs, causing symptoms. Resection of an IBC leads to an improvement in symptoms. We completely resected an IBC by performing median sternotomy and the symptom improved, and we described the clinical course.

**Case presentation:**

A 48-year-old man with palpitation and chest discomfort was referred to our institution. Chest computed tomography (CT) revealed a 42 × 35 × 32-mm cystic mass attached to the right pulmonary artery and right bronchus. Chest CT also revealed that this cystic mass compressed the superior vena cava, right superior pulmonary vein, and left atrium. Endobronchial ultrasonography transbronchial needle aspiration (EBUS-TBNA) revealed a mucinous fluid with high CA19-9 level (> 12,000 U/mL). We performed complete resection of the IBC that firmly adhered to the superior pulmonary vein and left atrium with cardiopulmonary bypass (CPB) using median sternotomy. The postoperative course was uneventful, and the patient was discharged on postoperative day 16 without major complications. The cystic mass was diagnosed as an IBC. He has been well without symptom of chest discomfort and any signs of recurrence for 18 months postoperatively.

**Conclusions:**

For complete resection of an IBC, CPB needs to be anticipated for difficulty in predicting firm adhesion to the heart and great vessels. Measuring the CA19-9 level in the sampling fluid with EBUS-TBNA can be useful for the preoperative IBC diagnosis.

## Background

Bronchogenic cysts (BCs) are congenital thoracic anomalies that develop from the ventral foregut during embryogenesis and typically develop in the pericarinal, paratracheal, and intrapulmonary regions, and occasionally in the pericardium [1]. BCs produce CA19-9 from their epithelium, and measuring the CA19-9 level of the intrapericardial BC (IBC) fluid can be useful for preoperative IBC diagnosis. An IBC compresses surrounding organs and causes symptoms such as chest pain, palpitation, cough, and dyspnea. BCs are usually benign, and their complete resection is controversial. However, by resecting an IBC, an improvement in symptom is expected. Other reasons for resection are to rule out malignancy and diagnose a malignant tumor while it is still resectable. Herein, we completely resected an IBC causing symptoms by using a cardiopulmonary bypass (CPB) with median sternotomy and described the clinical course.

## Case presentation

**Fig. 1:**
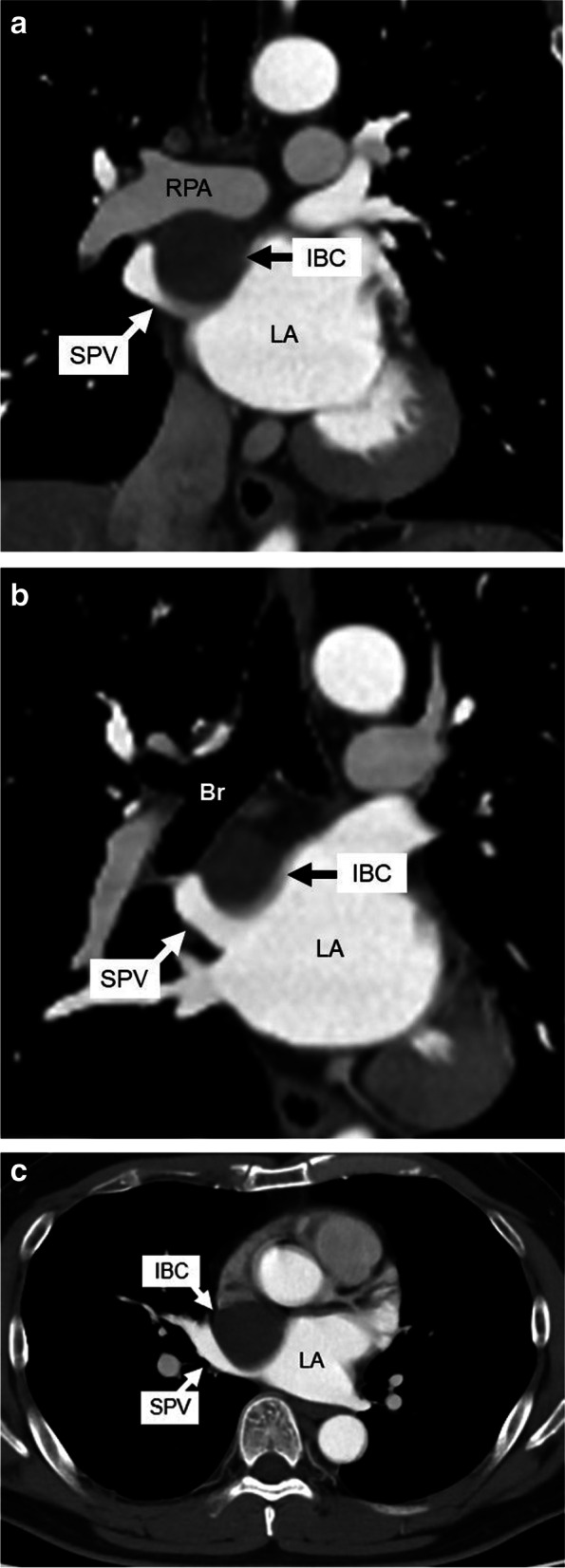
Coronal and axial view of preoperative chest CT. **a** A 42 × 35 × 32-mm cystic mass at the intrapericardium located under the RPA and compressing the SPV and LA. **b** The cystic mass attached to the right bronchus and compressing the SPV and LA. **c** The cystic mass compressing the SPV and LA

**Fig. 2:**
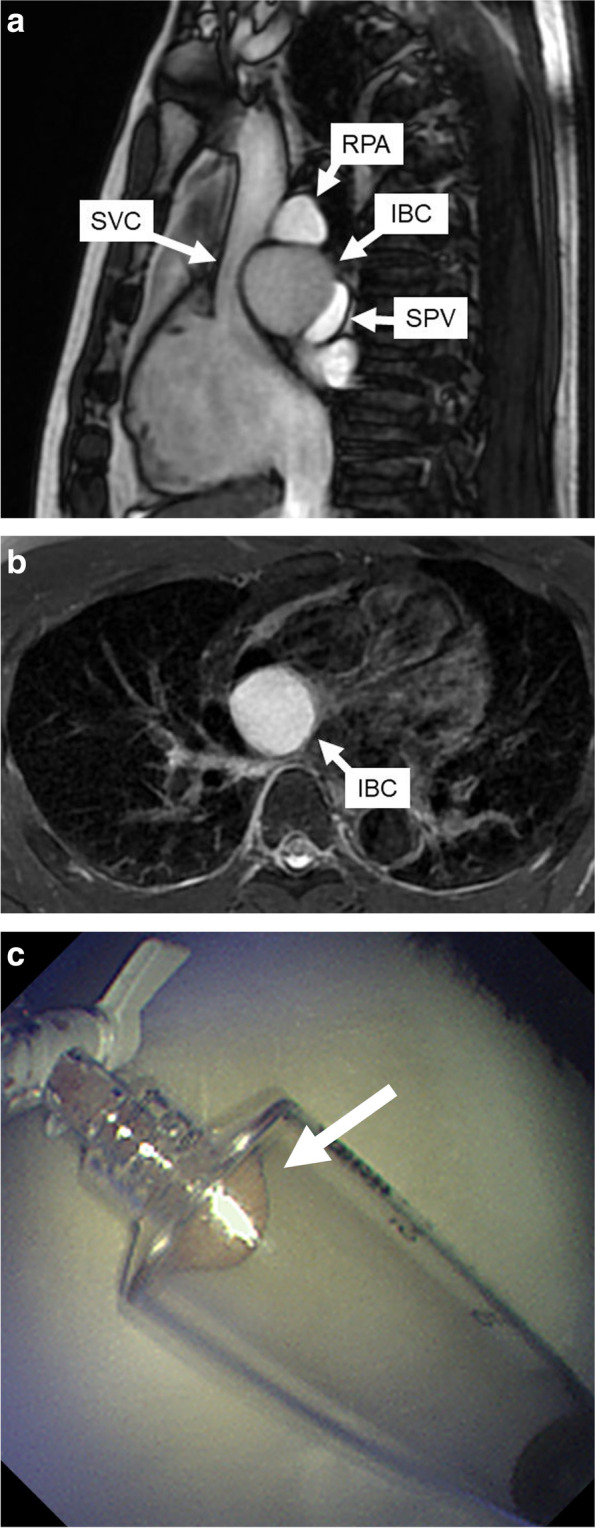
Sagittal and axial view of chest MRI, and sampling fluid by EBUS-TBNA. **a** The iso-intensity of the cystic mass on T1-weighted images compressing the SVC and SPV. **b** The high intensity of the cystic mass on T2-weighted images. **c** The yellowish brown mucinous fluid (white arrow)

**Fig. 3:**
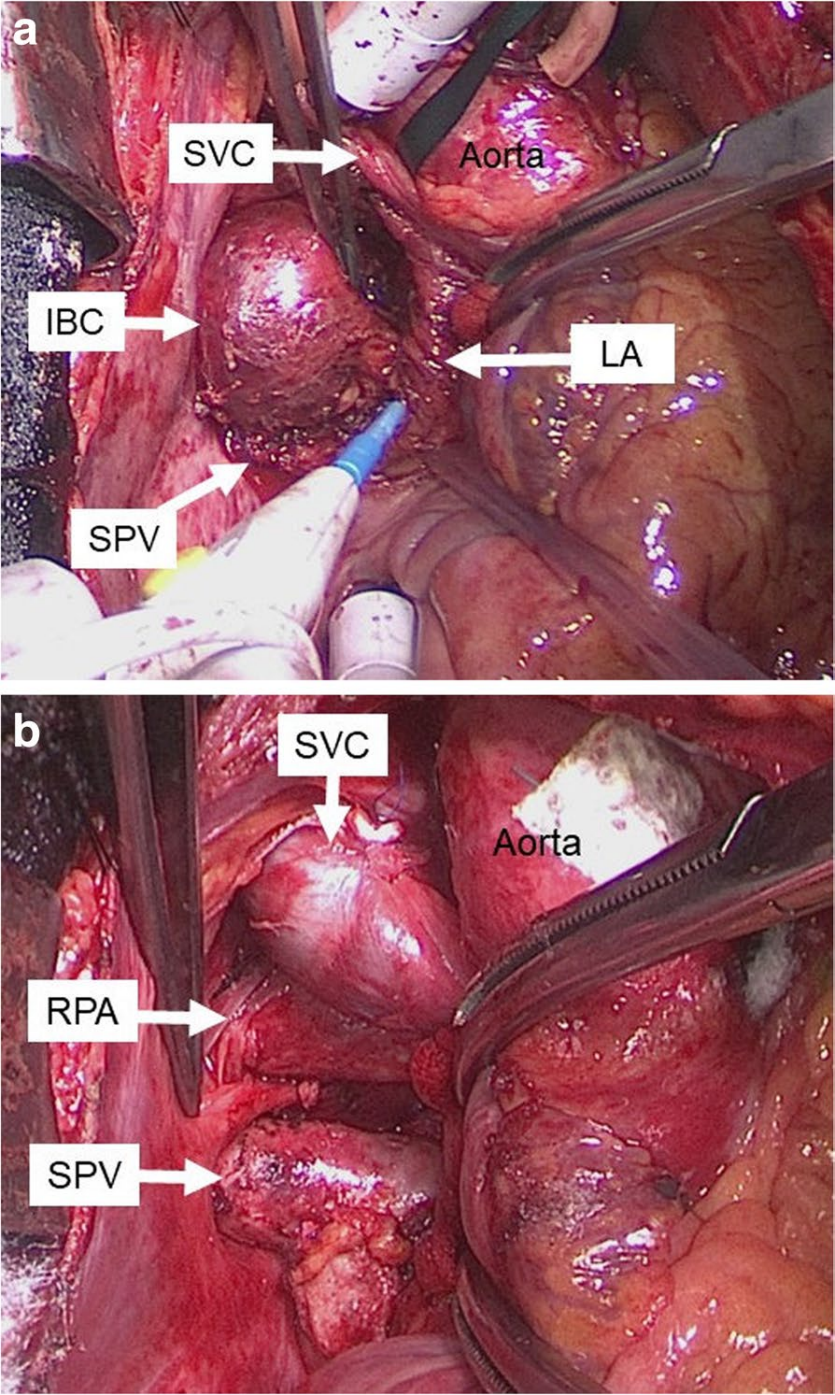
Intraoperative photograph. **a** The firm adhesion of the IBC to the SPV and LA. The IBC was resected using CPB. **b** The intrapericardium after resection of IBC

**Fig. 4:**
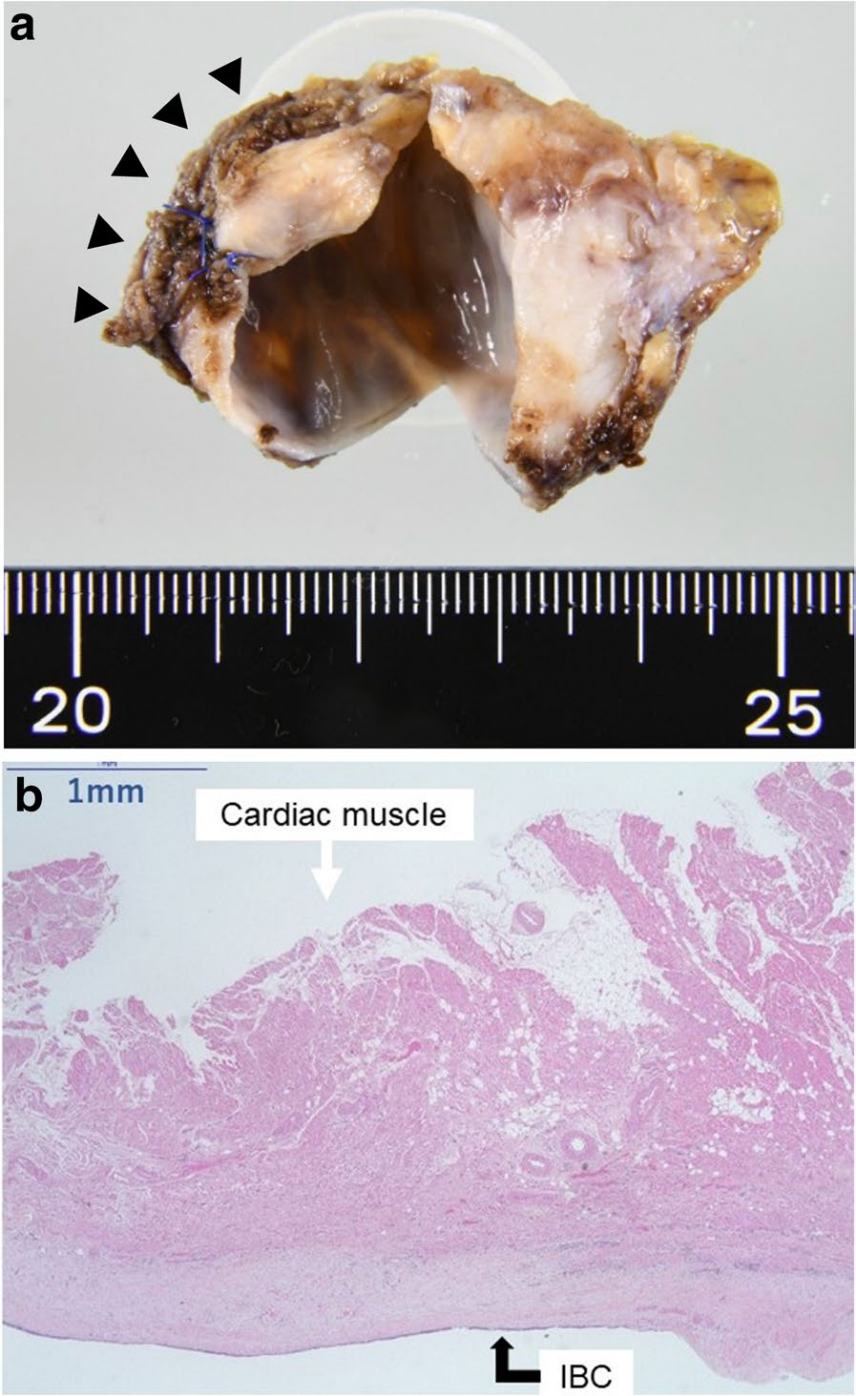
The resected specimens and the microscopical findings. **a** The cardiac muscle shown by black triangles resected with IBC. **b** No layer between the cardiac muscle and IBC. A scale bar indicates 1 mm

A 48-year-old man with palpitation and chest discomfort was diagnosed with paroxysmal atrial fibrillation (PAF) by portable electrocardiography. Then, he was referred to our hospital for ablation therapy. He regularly received anticoagulant and arrhythmia therapy with rivaroxaban and pilsicainide. Physical examination and vital signs were unremarkable. Heart rate was 60 bpm on 12-lead electrocardiography. Laboratory tests were within the reference change. Chest computed tomography (CT) revealed a 42 × 35 × 32-mm cystic mass attached to the right pulmonary artery (RPA) and right bronchus (Br). Chest CT also revealed that this cystic mass compressed the superior vena cava (SVC), right superior pulmonary vein (SPV), and left atrium (LA) (Fig. 1a–c). Magnetic resonance imaging (MRI) of the chest revealed that the mass had iso-intensity on T1-weighted images and high intensity on T2-weighted images (Fig. 2a, b). Endobronchial ultrasonography (EBUS) revealed low echoic lesion with foci of moderate echoic signal and transbronchial needle aspiration (TBNA) revealed yellowish brown mucinous fluid with high CA19-9 level (> 12,000 U/mL) (Fig. 2c). An IBC was suspected based on these findings, and a surgery for the complete resection of the cystic mass was planned. We used median sternotomy and taped the SVC and RPA in the pericardial space without adhesion. Then, the cyst was resected from the right Br using an energy device passing between the SVC and ascending aorta. However, owing to its firm adhesion to the SPV and LA (Fig. 3a). The partial CPB was initiated with cannulations to the ascending aorta and the superior and inferior vena cavae. Then, the firm adhesion part of SPV and LA was exfoliated with electrocautery, and complete resection of the cystic mass was performed without blood transfusion (Fig. 3b). The injured lesion of the LA in complete resection was repaired by non-absorbable monofilament with pledget. The operative time was 378 min, and the time of CPB was 55 min. Microscopic observation revealed the presence of pseudostratified ciliated epithelial cells, smooth muscle, and cartilage without malignancy, and an IBC was diagnosed, and the cardiac muscle tissue that was resected was found with IBC (Fig. 4a, b). The postoperative course was uneventful, and the patient was discharged on postoperative day 16 without major complications. He has been well with no symptom of chest discomfort and no PAF on the 24-h Holter electrocardiogram at 7 months postoperatively, and without signs of recurrence at 18 months.

## Discussion

IBC is a rare congenital lesion of BC. BCs are congenital lesions resulting from abnormal budding of the ventral foregut that develops between the 26th and 40th days of gestation. This abnormal bud subsequently differentiates into a fluid-filled, blind-ending pouch. Most cysts are located in the mediastinum, near the tracheal carina [2]. The abnormal buds are rarely displaced within the intrapericardium and separate from the tracheobronchial tree in IBC [1].

BCs are usually benign, and complete resection of BCs is controversial. To the best of our knowledge, excluding recurrence of IBCs, there are 12 reported cases of IBC resection in adults, including the present case in English [1, 3–11] (Table 1). Partial resection without CPB was mainly performed [3, 4, 6–8]. Complete resection with CPB was performed in only two cases [9, 11]. Therefore, it is significant to describe the surgical findings and clinical course of complete resection with CPB of IBC. Partial resection can cause recurrence, reoperation is required [12, 13], and carcinoma can coexist [14]. Complete surgical resection avoids recurrence and helps establish a diagnosis, alleviate symptoms, and prevent complications, such as pericardial effusion, cardiac tamponade, arrhythmias, heart compression, and acute pericarditis [6]. CPB via a sternotomy may be an over-indicated procedure, and generally, the video-assisted thoracoscopic surgery seems acceptable. However, a recurrence case using thoracotomy has been reported 19 years postoperatively [12], and the firm adhesion is predicted in redo to IBC recurrence, which will make it a difficult procedure. Therefore, we selected complete IBC resection using CPB.

**Table 1:** Clinical features of resected adult cases of IBC, including the present case in English

Case	Year	Age	Sex	Diameter (cm)	Approach	Adhesion (organ)	Surgery	CPB
1 [3]	1966	60	M	17.5	T	+(A,SVC,PA,PV)	PR	−
2,3 [1]	1975	45	F	12	Me	+(P)	CR	−
		50	M	12	Me	+(A)	CR	−
4 [4]	2003	34	F	6	T	ND	PR	−
5 [5]	2011	33	F	9.8	R	+(P)	CR	−
6 [8]	2011	39	F	5.7	T	+(A)	PR	−
7 [6]	2013	43	M	10	T	+(A, P)	PR	−
8 [9]	2014	39	F	9.4	Me	+(RCA, Ao)	CR	+
9 [10]	2014	25	M	8	Me	ND	CR	−
10 [7]	2016	42	F	9	T	+(GV)	PR	−
11 [11]	2021	66	F	10	Me	+(A、PV)	CR	+
Our case	-	48	M	4.5	Me	+(A、PV, SVC, PA)	CR	+

*ND* not described, *M* male, *F* female, *T* thoracotomy, *Me* median sternotomy, *R* robot, *P* pericardium, *A* atrium, *Ao* aorta, *RCA* right coronary artery, *PA* pulmonary artery, *PR* partial resection, *CR* complete resection, *GV* great vessel, *CPB* cardiopulmonary bypass

Thoracotomy is a daily procedure for general thoracic surgeons, and we can perform the procedure in a familiar field of view. However, the exposure of the great vessels and the heart is limited, and complete IBC resection is difficult at the risk of cardiac tissue injury and massive bleeding. Median sternotomy offers several advantages, including superb exposure of the heart and great vessel that surround an IBC, as well as ease of setting up CPB compared with thoracotomy although we need to mobilize the great vessels in the pericardial space. Although we can prevent the recurrence by firmly cauterizing the residual wall of the cyst with electrocautery, the heart and the great vessels attached to IBC can be injured and massive bleeding may arise by firm cauterization. Therefore, we performed complete resection of IBCs with median sternotomy exfoliating the firm adhesion part of SPV and LA with electrocautery using CPB. No previous reports show that preoperative graphic studies can predict the existence of adhesion between IBC and adjacent organs such as the heart and the great vessels. We are unable to accurately determine how cases with a need for CPB can be predicted and demarcated. There is no definite standard of preparing for CPB; this investigation is a challenge for the future. For complete resection of IBCs, to date, we need to anticipate CPB for difficulty in predicting the firm adhesion to the heart and great vessels. Our case had firm adhesion to the SPV and LA and CPB was set up. Microscopically, our case showed partial cardiac tissue that was partially resected in adhesion detachment to LA, and we reinforced this injured cardiac point using monofilament with pledget.

The attenuation of the contents of BCs can vary from that of water to soft tissue [2, 15]. The value of attenuation can be as high as 100 Hounsfield units if the cyst contains protein or calcium oxalate [15]. Most cysts that we classified as having soft-tissue attenuation on CT were hyperintense to cerebrospinal fluid on T2-weighted magnetic resonance (MR) images and isointense to skeletal muscle on T1-weighted MR images. These findings suggest that mucus and proteinaceous debris, not calcium, within the cyst are the most likely explanation for the increased attenuation observed on CT [2]. Bronchoscopy is a useful tool in the diagnosis of BCs, both for confirmation of the cystic nature of the lesion by EBUS and diagnosis by TBNA of the cyst fluid [16]. EBUS shows a hypoechoic (dark gray) or anechoic (black) lesion abutting the major airways and absent Doppler signal. Sometimes, BC is associated with a high CA 19-9 level for production of CA19-9 from the epithelium of BC [17, 18]. In this case, the CT and MR images revealed protein attenuation and intensity, and the CA19-9 level of the fluid in IBC was high. Therefore, we can make a presumptive diagnosis of BCs preoperatively. To the best of our knowledge, preoperative diagnosis of IBC using EBUS-TBNA has not been reported until now. Although therapy of BCs by TBNA is reported [16], in our case, we did not perform TBNA for therapy considering injury to the great vessels or atrium by the needle.

The majority of these cysts remain asymptomatic. However, depending on the size of the cyst and possible compression of adjacent cardiac structures, symptoms such as chest pain, cough, dyspnea or acute respiratory distress, atrial fibrillation, SVC obstruction, and spontaneous pneumopericardium have been predicted. IBC compresses the heart and causes complications. Some cases reported that resection of BCs improved PAF [11, 13, 19]. In our case, symptom and PAF improved via IBC resection.

Complete resection of IBCs that have firm adhesion to the surrounding organs, such as the heart and great vessels, requires CPB. Measuring the level of CA19-9 in the sampling fluid with EBUS-TBNA can be useful for the preoperative diagnosis of IBC. Symptom for IBC can improve by complete resection.

## Data Availability

Case report data and patient’s consent form are available.

## Abbreviations

CT: Computed tomography
MRI: Magnetic resonance imaging
PAF: Paroxysmal atrial fibrillation
IBC: Intrapericardial bronchogenic cyst;
CPB: Cardiopulmonary bypass
TBNA: Transbronchial needle aspiration
EBUS: Endobronchial ultrasonography
Br: Bronchus
RPA: Right pulmonary artery
SVC: Superior vena cava
SPV: Superior pulmonary vein
LA: Left atrium
CA19-9: Carbohydrate antigen 19-9
